# Predation by nematode-trapping fungus triggers mechanosensory-dependent quiescence in *Caenorhabditis elegans*

**DOI:** 10.1016/j.isci.2025.112792

**Published:** 2025-05-30

**Authors:** Tzu-Hsiang Lin, Han-Wen Chang, Rebecca J. Tay, Yen-Ping Hsueh

**Affiliations:** 1 Institute of Molecular Biology, Academia Sinica, Taipei 115, Taiwan; 2 Genome and Systems Biology Degree Program, Academia Sinica and National Taiwan University, Taipei 106, Taiwan; 3 Molecular Cell Biology, Taiwan International Graduate Program, Academia Sinica and Graduate Institute of Life Science, National Defense Medical Center, Taipei 114, Taiwan; 4 Department of Complex Biological Interactions, Max Planck Institute for Biology Tübingen, 72076 Tübingen, Germany

**Keywords:** behavioral neuroscience, biological sciences, neuroscience

## Abstract

Animals exhibit diverse behavioral adaptations to predation, driving coevolution across the Tree of Life. Using *Caenorhabditis elegans* as a genetic model organism, we investigated how nematodes respond to predation by the nematode-trapping fungus *Arthrobotrys oligospora*. Fungal trapping induces quiescence in *C. elegans*, characterized by a rapid cessation of pharyngeal pumping and movement. This quiescence is regulated by the activation of sleep-promoting neurons Anterior Lateral neuron A (ALA) and Ring Interneuron S (RIS), with genetic analyses demonstrating that ALA is essential for inhibiting pharyngeal pumping while both ALA and RIS contribute to movement cessation. Mechanosensation and epidermal growth factor receptor (EGFR) signaling are critical to these responses, demonstrating how prey neurophysiology responds to a sessile predator. These findings shed light on the neuronal and molecular mechanisms of stress-induced behaviors, revealing how fungal traps trigger behavioral responses in prey and advance our understanding of predator-prey dynamics.

## Highlights

• Nematode-trapping fungus *A. oligospora* induces quiescence in *C. elegans*
• Fungal trapping activates sleep-promoting ALA and RIS neurons in *C. elegans*
• ALA activation inhibits pharyngeal pumping, whereas RIS and ALA suppress movement
• Mechanosensory and EGFR signaling pathways mediate trapping-induced quiescence

## Graphical abstract

**Figure:**
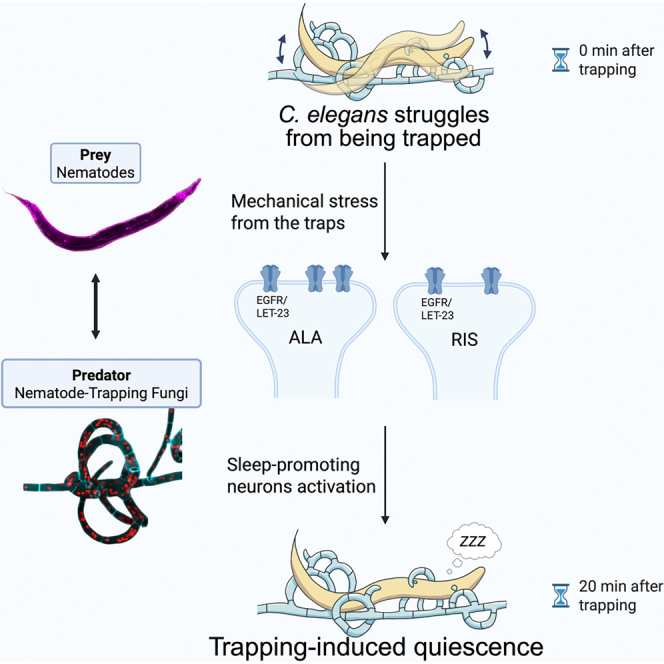

## Introduction

Predator-prey interactions are fundamental ecological processes that drive the coevolution of both predators and their prey. Predation exerts significant selective pressure, compelling prey to adopt behavioral and physiological adaptations such as reduced foraging, suppressed reproduction, and decreased motility to mitigate risks of being captured or killed.^1^^,^^2^^,^^3^^,^^4^ These interactions have shaped diverse adaptive strategies over evolutionary timescales, influencing the physiology, behavior, and survival of organisms, yet the underpinning molecular and cellular mechanisms that govern predator-prey interaction and coevolution are largely unexplored in any given system due to lack of proper genetic models.

The nematode *Caenorhabditis elegans* serves as an exceptional model for studying predator-prey interactions and coevolution at molecular, cellular, and behavioral levels. Its natural interactions with predators, such as the nematode-trapping fungus *Arthrobotrys oligospora*, coupled with its comprehensive genetic toolkit, provide unique opportunities to uncover the mechanisms underlying these complex dynamics.^5^ Molecular clock and fossil evidences indicate that this predator-prey interaction has persisted for over 400 million years.^6^ Our previous work demonstrated that nematodes and nematode-trapping fungi coexist in diverse soil environments, with sympatric distribution in over 60% of collected soil samples, and that *C. elegans* co-habitats with the nematode-trapping fungus *A. oligospora.*^5^ Efforts have been made to make *A. oligospora* highly genetically tractable, and thus *A. oligospora* and *C. elegans* represent a unique predator-prey system in which one can genetically dissect the molecular mechanism of the interactions from both sides. For example, it has been shown that *A. oligospora* employs sophisticated strategies to capture nematodes, including the production of volatile organic compounds (VOCs) that mimic nematode food and sex cues,^7^ responding to nematode pheromones, ascarosides,^8^ via G-protein-coupled receptors,^9^^,^^10^ triggering trap formation through conserved signaling pathways such as the mitogen-activated protein kinases (MAPKs) and cAMP-PKA pathways.^11^^,^^12^ While these fungal adaptations have been characterized, the response of *C. elegans* to *A. oligospora* predation remains less understood.

*C. elegans* encounters a variety of abiotic and biotic stresses, such as temperature fluctuations, osmotic shock, starvation, infections, and predation.^13^^,^^14^ Notably, many environmental stresses induce a sleep-like state in *C. elegans*.^15^ This stress-induced sleep is regulated by two peptidergic sleep-promoting interneurons, Anterior Lateral neuron A (ALA) and Ring Interneuron S (RIS), and involves the epidermal growth factor receptor (EGFR) signaling pathway, which is crucial for the stress response.^15^^,^^16^^,^^17^^,^^18^^,^^19^ This stress-induced sleep mechanism is conserved across diverse taxa, including nematodes, flies, zebrafish, and mice, and is known to enhance survival during adverse conditions.^20^^,^^21^^,^^22^^,^^23^ Recent findings suggested that antimicrobial peptides (AMPs) upregulated in response to pathogen infection and wounding also regulate sleep in *C. elegans* and other species, facilitating communication between peripheral tissues and the nervous system.^22^^,^^24^

In this study, we investigated the behavioral, neuronal, and physiological response of *C. elegans* to *A. oligospora* predation. We found that predation induces a quiescent state in *C. elegans* that depends on the activation of the sleep-promoting neurons ALA and RIS. Additionally, we identified a role for mechanosensation in mediating behavioral quiescence that is triggered by *A. oligospora* predation. Our findings suggest that *A. oligospora* predation exerts mechanical stress on *C. elegans* that leads to a quiescent state, providing new insights into the molecular and neuronal mechanisms underlying prey responses to predation stress.

## Results

### *A. oligospora* predation induces behavioral quiescence in *C. elegans*

**Figure 1:**
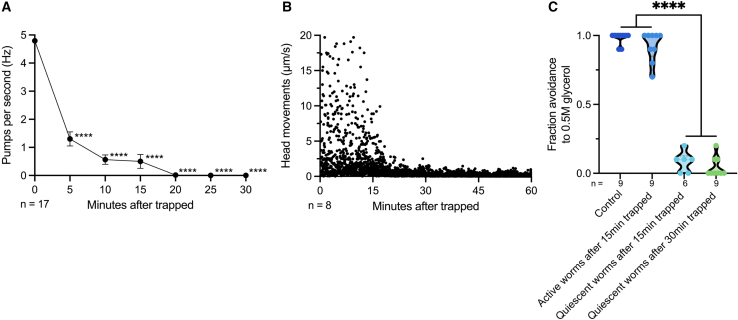
*A. oligospora* predation-induced quiescence in *C. elegans* (A) Pharyngeal pumping rates of WT nematodes after *A. oligospora* trapping. Mean ± SEM. One-Way ANOVA, Tukey's multiple comparison test, comparing to time 0; ∗∗∗∗*p* < 0.0001. (B) Head movements of WT nematodes after *A. oligospora* trapping. Each dot represents an individual movement recorded at each time point. (C) Response of *WT* nematodes to an aversive 0.5 M glycerol stimulus at different time points and behavior states after *A. oligospora* trapping. One-way ANOVA, Tukey’s multiple comparison test; ∗∗∗∗*p* < 0.0001.

To investigate the response of *C. elegans* to predation by nematode-trapping fungi, we observed the behavior of nematodes trapped by *A. oligospora*. When placed on a fungal lawn, nematodes were quickly captured upon contact with the adhesive traps and were unable to escape. Initially, the trapped nematodes exhibited vigorous struggling behavior, including increased thrashing, which appeared to be escape attempts. However, this activity was unsuccessful. Notably, within approximately 15–20 min, most trapped nematodes became largely quiescence (Video S1). To further characterize this behavior, we measured and quantified the three features of quiescence in *C. elegans*: pharyngeal pumping, movement, and response to external stimuli. We found that *A. oligospora*-trapping significantly inhibited the pharyngeal pumping, which decreased from 4.8 Hz to 1.3 Hz within the first 5 min and stopped entirely after about 20 min (Figure 1A). Analysis of head movements showed that most nematodes struggled continuously for around 20 min before entering a quiescent state (Figure 1B). We then tested the response to an aversive stimulus by applying glycerol to the head of the animals and monitored its behavior.^25^ Control animals and those still struggling after *A. oligospora* trapping exhibited a strong avoidance response to glycerol (Figure 1C). In contrast, *C. elegans* that had entered a quiescent state after 15 or 30 min of trapping showed a marked reduction in the avoidance behavior (Figure 1C). Overall, our results demonstrate that predation by *A. oligospora* induces a quiescence state in *C. elegans*, characterized by inhibition of pharyngeal pumping, cessation of movement, and reduced responsiveness to external stimuli.

### The sleep-promoting neurons ALA and RIS are activated in response to *A. oligospora* trapping

**Figure 2:**
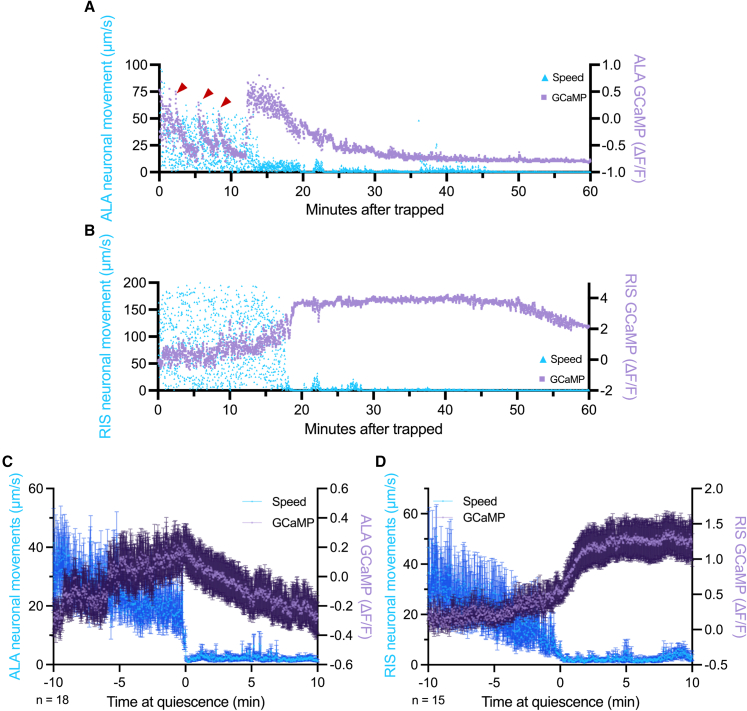
Sleep-promoting neurons ALA and RIS are activated in response to *A. oligospora* trapping (A) Representative calcium trace and movement of ALA in a wild-type animal after trapping, showing transient activation followed by maximal activation coinciding with movement quiescence as represented by neuronal movement. Red arrows indicate the early, small transient activations of the ALA neuron. (B) Representative calcium trace and movement of RIS in a wild-type animal after trapping, showing activation coinciding with movement quiescence as represented by neuronal movement. (C) Average neuronal calcium activity and movement of ALA aligned to the onset of movement quiescence, showing data from 10 min before to 10 min after quiescence. Mean ± SEM. (D) Average neuronal calcium activity and movement of RIS aligned to the onset of movement quiescence, showing data from 10 min before to 10 min after quiescence. Mean ± SEM.

In *C. elegans*, the sleep-promoting neurons ALA and RIS are essential for the induction of stress-induced sleep.^16^^,^^26^ To determine whether quiescence induced by *A. oligospora* trapping also involves these neurons, we monitored their activity in trapped nematodes by measuring calcium levels using GCaMP imaging. Shortly after trapping, ALA exhibited small, transient activation during the first 10 min, while the animals were still struggling and pumping was rapidly inhibited. ALA then reached maximal activation 15 to 20 min post-trapping, at which point *C. elegans* stopped struggling and entered a quiescence state. After entering quiescence, ALA calcium levels gradually declined (Figure 2A). In contrast, RIS displayed a distinct calcium activity pattern. During the initial struggling phase, RIS maintained low calcium level for the first 15 min. RIS then reached the maximal activation, corresponding with the onset of movement quiescence (Figure 2B). To further correlate neuronal activity with behavior states, we aligned individual calcium recordings with the onset of movement quiescence. ALA’s peak activation coincided with movement cessation and decreased thereafter, resulting in a low correlation coefficient (Spearman’s rank correlation of 0.1325) due to transient peaks and a decline after quiescence onset (Figure 2C). In contrast, RIS activation strongly coincided with movement quiescence, maintaining high calcium activity during the entire quiescent stage, resulting in a stronger negative correlation (ρ = −0.5648) (Figure 2D). These findings suggest that ALA and RIS activation in response to trapping contributes to the quiescent behavior in *C. elegans*, with their distinct activity patterns potentially coordinating the timing of pumping inhibition and movement cessation observed in *A. oligospora*-trapped nematodes.

### The sleep-promoting neurons regulate pharyngeal pumping and movement inhibitions

**Figure 3:**
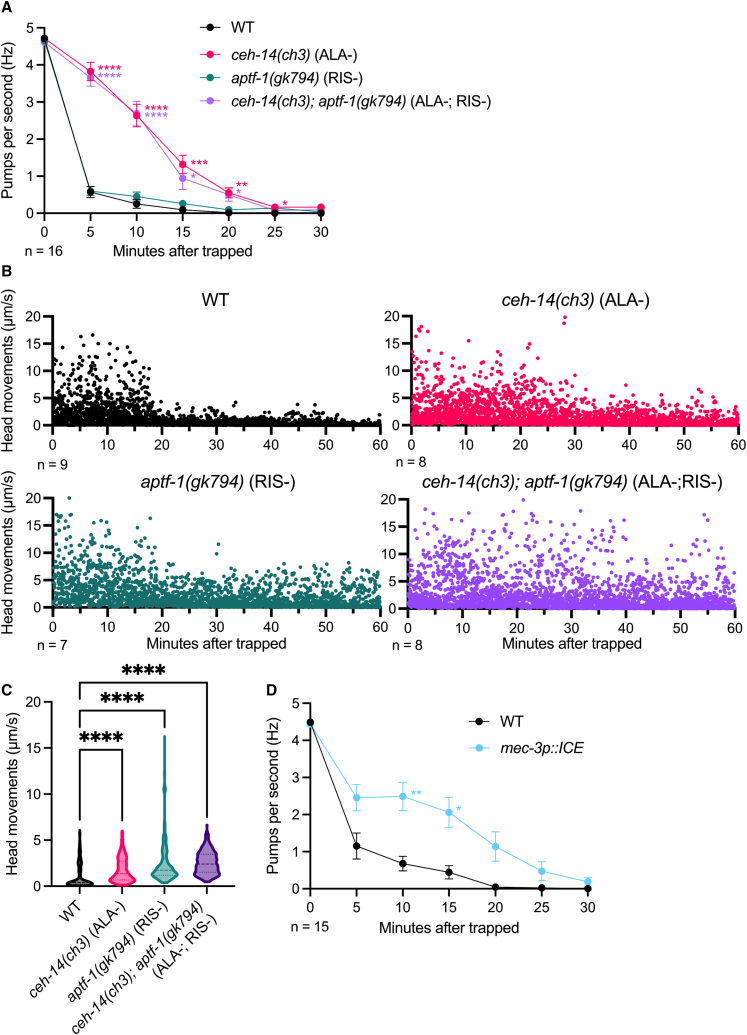
Trapping-induced quiescence is regulated by sleep-promoting neurons and mechanosensory neurons (A) Pharyngeal pumping rates in wild-type, ALA-deficient, RIS-deficient, and ALA/RIS-deficient mutants after *A. oligospora* trapping. (B) Head movements in wild-type, ALA-deficient, RIS-deficient, and ALA/RIS-deficient mutants after *A. oligospora* trapping. Each dot represents an individual movement recorded at each time point. (C) Distribution of head movements in wild-type, ALA-deficient, RIS-deficient, and ALA/RIS-deficient mutants (B). (D) Pharyngeal pumping rates in mutants with genetically ablated mechanosensory neurons after *A. oligospora* trapping. (A and D) Mean ± SEM; two-way ANOVA, Šidák’s multiple comparison test, comparing strain effects at each time point; ∗*p* < 0.05, ∗∗*p* < 0.01, ∗∗∗*p* < 0.001, ∗∗∗∗*p* < 0.0001. (C) Kruskal-Wallis test with Dunn’s correction; ∗∗∗∗*p* < 0.0001. See also Figures S1 and S2.

Studies have shown that the LIM homeodomain protein CEH-14 is essential for ALA-specific gene expression and axon outgrowth,^27^ whereas the AP2 transcription factor APTF-1 is necessary for RIS function.^16^ To examine the roles of ALA and RIS neurons in pharyngeal pumping inhibition and movement cessation upon *A. oligospora* trapping, we compared the behavior of *ceh-14(ch3)* (ALA-deficient) and *aptf-1(gk794)* (RIS-deficient) mutants to that of wild-type nematodes. In wild-type animals, pharyngeal pumping rate dropped sharply from 4.7 Hz to 0.6 Hz within the first 5 min after trapping (Figure 3A). In contrast, *ceh-14* mutants exhibited only a modest reduction in pumping rate, from 4.7 Hz to 3.8 Hz during the same period, and their overall decrease in pumping rate was more gradual compared to wild-type (Figure 3A). Meanwhile, the *aptf-1* mutant showed a similar degree of pumping inhibition as wild-type animals, and the *ceh-14 aptf-1* double mutant did not further reduce pumping beyond the inhibition observed in the *ceh-14* mutant alone (Figure 3A). These findings, together with ALA’s early calcium activation pattern observed during the initial trapping period (Figure 2A), suggest that ALA neurons are responsible for transmitting the signals that inhibit pharyngeal pumping upon *A. oligospora* predation, whereas RIS is not involved in trapping-induced pharyngeal pumping inhibition (Figure 3A).

We then tested the roles of these sleep-promoting neurons in the cessation of movement induced by *A. oligospora* predation. We measured the head movement of wild-type, *ceh-14*, *aptf-1*, and *ceh-14; aptf-1* mutants after trapping. Unlike the results for pumping inhibition, both ALA- and RIS-deficient mutants were more active than wild-type nematodes after *A. oligospora* trapping (Figures 3B and 3C). These data suggest that both ALA and RIS neurons play a role in regulating the movement cessation triggered by *A. oligospora* trapping.

### Mechanosensory neurons regulate trapping-induced pumping inhibition

To identify which signals from *A. oligospora* traps initiate the quiescence response in *C. elegans*, we hypothesized that the mechanical immobilization of nematodes by trap adhesion might serve as a key stimulus. Supporting this idea, we observed trapped *C. elegans* exhibited robust egg-laying (Video S1), a behavior known to be mechanically induced.^28^ Additionally, previous study has shown that physical confinement within a microfluidic device can induce a sleep-like state in *C. elegans* via the mechanosensory pathway and RIS neuron activation.^29^ To test this hypothesis, we mimicked the immobilizing effect of trap adhesion by glue-immobilization with WormGlu (GluStitch Inc.), a dental-grade cyanoacrylate glue designed for electrophysiology recordings in nematodes. Nematodes were briefly immobilized with cold treatment to apply the glue and then allowed to recover at room temperature. After recovery, while control animals resumed pharyngeal pumping within five minutes, glued nematodes failed to recover pumping (Figure S1A). Although potential glue toxicity cannot be ruled out, this result implies that physical restraint with WormGlu may inhibit pharyngeal pumping.

To further define the role of the mechanosensory pathway in *A. oligospora*-induced quiescence, we generated a mutant strain in which key mechanosensory neurons (ALML/R, AVM, FLP, PLML/R, PVD, and PVM) were genetically ablated by expressing human caspase ICE under the *mec-3* promoter. Upon trapping by *A. oligospora*, these mechanosensory-deficient mutants exhibited a milder reduction in pharyngeal pumping compared to wild-type animals (Figure 3D). These findings suggest that the mechanosensory pathway partially contributes to trapping-induced quiescence.

Next, we investigated whether mechanosensory pathways are involved in activating sleep-promoting neurons. Using calcium imaging, we monitored ALA and RIS activity in mechanosensory-deficient mutants (with 10 of the 30 mechanosensory neurons ablated) following *A. oligospora* trapping. ALA activity in the mutants during the initial 15 min differed from that in wild-type animals. Specifically, the transient peaks characteristic of wild-type ALA activity was significantly reduced in the mechanosensory-deficient mutants (Figures S2A and S2C). Although RIS activity was also reduced, this reduction was not statistically significant (Figures S2B and S2C). In wild-type animals, the median number of ALA peaks greater than 10% of the amplitude range and lasting more than 10 s was 12.5, whereas in mechanosensory-deficient mutants, it dropped to three peaks (Figure S2D). Despite this difference, the area under the curve for peak activity remained consistent between wild-type and mutant animals (Figure S2E). The reduced early activity in ALA neurons, combined with the functional analyses and delayed pumping inhibition in mechanosensory-deficient mutants, suggests that mechanosensation is necessary for initiating pumping quiescence. ALA has been identified as a high-threshold mechanosensory neuron, potentially contributing to this process through its mechanosensory property.^30^ However, the partial reduction in ALA and RIS activity implies that additional mechanosensory neurons or potentially chemical cues from *A. oligospora* may act alongside to mechanical stress to induce quiescence in *C. elegans*.

### Epidermal growth factor receptor signaling pathways in ALA and RIS are required in trapping-induced quiescence

**Figure 4:**
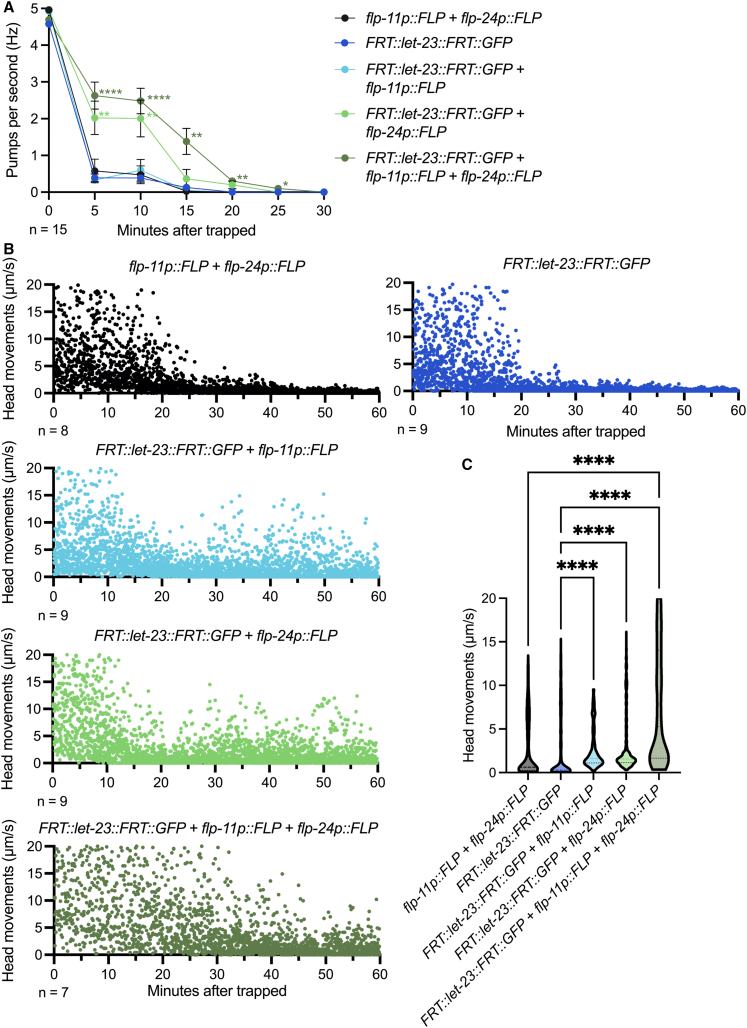
EGFR signaling in ALA and RIS is required for trapping-induced quiescence (A) Pharyngeal pumping rates following *A. oligospora* trapping in FLPase-only control, *let-23(frt)* control, ALA-specific *let-23* knockdown, RIS-specific *let-23* knockdown, and ALA/RIS dual *let-23* knockdown animals. Mean ± SEM; two-way ANOVA with Šidák’s multiple comparison test to compare strain effects at each time point; ∗*p* < 0.05, ∗∗*p* < 0.01, ∗∗∗*p* < 0.001, ∗∗∗∗*p* < 0.0001. (B) Head movements following *A. oligospora* trapping in control animals and conditional *let-23* knockdown mutants. Each dot represents an individual movement recorded at each time point. (C) Distribution of head movements for each genotype from (B). Kruskal-Wallis test with Dunn’s correction; ∗∗∗∗*p* < 0.0001.

In *C. elegans*, stress-induced sleep involves the EGFR signaling pathway.^26^^,^^31^ The *C. elegans* homolog of EFGR, LET-23, is expressed in the sleep-promoting neurons ALA and RIS.^32^ Previous studies demonstrated that conditional knockdown of *let-23* using FLPase-induced recombination can be employed to investigate its role in stress responses, such as heat shock.^32^ Here, we used the neuron-specific promoters *flp-24* and *flp-11* to drive FLP recombinase expression in ALA and RIS neurons, enabling targeted knockdown of *let-23*. Disruption of the EGFR signaling in ALA resulted in prolonged pharyngeal pumping, whereas RIS-specific knockdown showed pumping inhibition similar to the FLPase-only and *FRT::let-23::FRT* control strains (Figure 4A). Consistent with the results from neuronal deficient mutants (Figure 3), both ALA- and RIS-specific *let-23* knockdown strains exhibited altered movement (Figures 4B and 4C). Together, these findings further support the conclusion that the EGFR signaling in ALA and RIS plays a critical role in mediating quiescence in response to *A. oligospora* predation.

## Discussion

*C. elegans* responds to predation by the nematode-trapping fungus *A. oligospora* by entering a quiescent state with reduced pumping and movement. This quiescence is driven by sleep-promoting neurons ALA and RIS, in conjunction with the mechanosensory and EGFR signaling pathways, suggesting that both mechanical stress and sleep-related mechanisms contribute to this behavior. These findings illustrate a multifaceted response in *C. elegans* that integrates behavioral quiescence, underscoring the complexity of its strategies to counteract predation stress.

High-resolution time-lapse imaging reveals that during the fungal predation process, *A. oligospora* begins forming an infection bulb and penetrate the nematode cuticle about 30–40 min after initial adhesion.^33^ This observation permits two non-exclusive interpretations. Stress-induced sleep could be an intrinsic damage response that suppresses locomotion, thereby improving the nematode’s survival chances under stress. Conversely, immobilization stabilizes the prey and thereby benefits the fungus by preventing prey escape from adhesive traps to increase predation success. Currently, our experimental system cannot discriminate between these scenarios because the optimized adhesive traps in the laboratory capture *C. elegans* at 100% efficiency, and no escape response was observed. Consequently, we cannot directly test reversibility of quiescence or measure survival, escape rates, or fungal growth in sleep-deficient mutants. We therefore present the co-option hypothesis as speculative and explicitly acknowledge the equally plausible adaptive model. Future work is required to quantify variable such as time-to-death, fungal hyphal expansion, and infection success in stress-induced sleep-deficient animals and thereby resolve whether trap-induced quiescence ultimately serves prey, predator, or both.

Stress-induced sleep is a well-documented phenomenon across diverse species.^20^^,^^21^^,^^22^^,^^23^ In *C. elegans*, quiescent states triggered by bacterial toxins, heat shock, or wounding are mediated by the EGFR pathway and sleep-promoting neurons such as ALA and RIS.^15^^,^^24^^,^^32^ These deleterious stresses cause cellular damage, which in turn induces stress-induced sleep to promote survival.^34^ Sleep deprivation reduces survival rates, but RIS activation alone is sufficient to improve survival by upregulating protective gene expression.^35^ Similarly, epidermal wounding triggers an innate immune response that upregulates antimicrobial peptides, such as NLPs (neuropeptide-like proteins) and CNCs (caenacins), which induce stress-induced sleep through the EGFR pathway.^24^ In contrast, a recent study has shown the nematode-trapping fungus, *Arthrobotrys flagrans*, induces the expression of *nlp-27*, leading to neurodegeneration.^36^

Our study demonstrated that *A. oligospora* predation engages the same stress-induced sleep neural circuitry through EGFR signaling in ALA and RIS. The neurons ALA and RIS exhibit distinct temporal activation patterns, with ALA showing transient depolarization within 5 min and RIS reaching peak activity at 20 min. This temporal dynamic is consistent with previous observations of heat-shock-induced quiescence, where ALA is thought to act upstream of RIS.^32^ However, the precise upstream signals that activate ALA and RIS during fungal trapping remain unclear. Recent findings have shown that *A*. *flagrans* secretes a virulence factor NipA that interferes *C. elegans* cuticle integrity and facilitates fungal penetration.^37^ Notably, the newly identified EGFR ligand SISS-1, which is essential for stress-induced sleep, is released from damaged tissue via the shedding by ADM-4 metalloprotease and further activates the EGFR LET-23 on sleep-promoting neurons.^31^ Thus, it is possible that mechanical stress and cuticular damages triggered by *A. oligospora* trapping might lead to SISS-1 release and activate EGFR signaling in ALA and RIS to induce quiescence.

Our study revealed that disrupting sleep circuits in *C. elegans* does not entirely abolish quiescence during fungal predation. Although these circuits are critical for initiating quiescence, additional mechanisms likely contribute to maintaining the nematode in this vulnerable state. We hypothesize that after capturing *C. elegans* using adhesive nets, *A. oligospora* secretes effectors to sustain quiescence. Supporting this, time-course transcriptional profiling of *A. oligospora* revealed a significant upregulation of secretion-related genes following nematode trapping.^38^ Similarly, studies on *A*. *flagrans* identified a fungal effector, CyrA, as critical for full virulence.^39^ *A. flagrans* also induces *nlp-27* upregulation, leading to neurodegeneration and paralysis.^36^ However, *nlp-27* appears to be only one of several factors involved, suggesting a multi-mechanistic regulation of nematode paralysis during fungal infection.^36^

Mechanosensory neurons, which constitute approximately 10% of the *C. elegans* nervous system,^40^^,^^41^ are essential for detecting physical stimuli. A study on another nematode-trapping fungus, *Drechslerella doedycoides*, which captures nematodes with constricting hyphal rings, demonstrated that nematodes exhibit a rapid reversal response upon contact with *D. doedycoides* traps, enabling escape behavior.^42^ In the touch-insensitive *mec-4* mutant, this escape response is impaired, resulting in higher capture rates.^42^ Our findings further reveal that mechanosensation contributes to trapping-induced quiescence, highlighting its dual role in escape behavior and quiescence induction.

Collectively, our results demonstrate that predation by the nematode-trapping fungus *A. oligospora* induces quiescence in *C. elegans* through the interplay of mechanosensory and sleep-promoting neurons. These findings shed light on the intricate neuronal and genetic mechanisms underlying the *C. elegans* response to fungal predation and provide broader insights into predator-induced behavioral adaptations in animals.

### Limitations of the study

A limitation of the experimental system is that trapped *C. elegans* cannot be recovered without physical damage. Consequently, it is not possible to assess the reversibility of the observed quiescent state or its potential adaptive significance. Additionally, our experiments utilized only one predator strain *A. oligospora* TWF154 and a laboratory prey strain *C. elegans* CGC1, raising the question of whether conserved neuronal and molecular mechanisms occur with other predatory fungi, wild isolates of *C. elegans*, or in natural soil ecosystems. Finally, our behavioral measures including pharyngeal pumping and head movement did not capture broader potential physiological responses and long-term survival outcomes. As a result, it is uncertain how this quiescent state affects fitness under predation stress.

## Resource availability

### Lead contact

Further information and requests for resources and reagents should be directed to and will be fulfilled by lead contact, Yen-Ping Hsueh (yphsueh@tuebingen.mpg.de).

### Materials availability

Strains and plasmids generated in this study are available upon request.

### Data and code availability

• All data reported in this paper will be shared by the lead contact upon request.
• This paper does not report the original code.
• Any additional information required to reanalyze the data reported in this paper is available from the lead contact upon request.

## Acknowledgments

We thank Dr. Henrik Bringmann, Dr. Chun-Liang Pan, and Dr. Chun-Hao Chen for sharing nematode strains. We thank Dr. Chun-Liang Pan and Dr. Ralf Sommer for helpful discussions of this manuscript. T.-H.L. is a member of the International Max Planck Research School “From Molecules to Organisms.” Some strains were provided by the CGC, which is funded by NIH Office of Research Infrastructure Programs (P40 OD010440). We thank WormBase. This work was supported by the 10.13039/501100001869Academia Sinica Investigator Award AS-IA-111-L02 to Y.-P.H. and the 10.13039/501100004189Max Planck Society. We would like to thank EMBO Global Investigator Network and Young Investigator Program for supporting Y.-P.H.

## Author contributions

Conceptualization, T.-H.L., H.-W.C., and Y.-P.H.; formal analysis, T.-H.L. and H.-W.C.; investigation, T.-H.L. and H.-W.C.; writing—original draft, T.-H.L., R.J.T., and Y.-P.H.; writing—review & editing, T.-H.L., R.J.T., and Y.-P.H.; visualization, T.-H.L.; supervision, Y.-P.H.; funding acquisition, Y.-P.H.

## Declaration of interests

The authors declare no competing interests.

## Declaration of generative AI and AI-assisted technologies

During the preparation of this work, the authors used ChatGPT-4o to enhance the fluency and clarity of the writing. After using this tool or service, the authors reviewed and edited the content as needed and take full responsibility for the content of the publication.

## STAR★Methods

### Key resources table

**Table:** 

REAGENT or RESOURCE	SOURCE	IDENTIFIER
**Bacterial and virus strains**		

*E. coli*: Strain OP50	*Caenorhabditis Genetics Center*	RRID:WB-STRAIN:WBStrain00041969

**Chemicals, peptides, and recombinant proteins**		

WormGlu	GluStitch	Lot: 0119

**Experimental models: Organisms/strains**		

Wild-type *Arthrobotrys oligospora*	Yang et al.^5^	TWF154
Wild-type *Caenorhabditis elegans*	*Caenorhabditis Genetics Center*	CGC1
*C. elegans: yphEx334[let-23p::GCaMP6s::unc-54 3′UTR +unc-122p::dsRed::unc-54 3′UTR]*	This paper	TWN4731
*C. elegans: goeIs304[flp-11p::SL1-GCaMP3.35-SL2::mKate2-unc-54-3′UTR, unc-119(+)]*	Wu et al.^43^	HBR1361
*C. elegans: ceh-14(ch3) X*	*Caenorhabditis Genetics Center*	TB528
*C. elegans: aptf-1(gk794) II*	Turek et al.^16^	HBR227
*C. elegans: aptf-1(gk794) II; ceh-14(ch3) X* Crossed TB528 with HBR227	This paper	TWN4678
*C. elegans: yphIs3[myo-2p::GCaMP6s::unc-54 3′UTR]*	Lee et al.^44^	TWN1915
*C. elegans: yphIs3[myo-2p::GCaMP6s::unc-54 3′UTR]; aptf-1(gk794) II*	This paper	TWN4679
*C. elegans: yphIs3[myo-2p::GCaMP6s::unc-54 3′UTR]; ceh-14(ch3) X*	This paper	TWN4680
*C. elegans: yphIs3[myo-2p::GCaMP6s::unc-54 3′UTR]; aptf-1(gk794) II; ceh-14(ch3) X*	This paper	TWN4681
*C. elegans: yphIs25[mec-3p::ICE +myo-2p::mCherry]* UV integrated of JPS278, Outcross 4X	Russell et al.^45^ and this paper	TWN4732
*C. elegans: yphIs25[mec-3p::ICE +myo-2p::mCherry]; goeIs304[flp-11p::SL1-GCaMP3.35-SL2::mKate2-unc-54-3′UTR, unc-119(+)]*	This paper	TWN4779
*C. elegans: yphIs25[mec-3p::ICE +myo-2p::mCherry]; yphEx334[let-23p::GCaMP6s::unc-54 3′UTR +unc-122p::dsRed::unc-54 3′UTR]*	This paper	TWN4780
*C. elegans: let-23(zh131[FRT::let-23::FRT::GFP::LoxP::FLAG::let-23]) II.*	Konietzka et al.^32^	AH5059
*C. elegans: yphEx344[flp-11p::FLP::unc-54 3′UTR +flp-24p::FLP::unc-54 3′UTR +unc-122p::GFP::unc-54 3′UTR]; let-23(zh131[FRT::let-23::FRT::GFP::LoxP::FLAG::let-23]) II.*	This paper	TWN4909
*C. elegans: yphIs3[myo-2p::GCaMP6s::unc-54 3′UTR]; let-23(zh131[FRT::let-23::FRT::GFP::LoxP::FLAG::let-23]) II.*	This paper	TWN4925
*C. elegans: yphEx349[flp-11p::FLP::unc-54 3′UTR +flp-24p::FLP::unc-54 3′UTR +unc-122p::GFP::unc-54 3′UTR]*	This paper	TWN5106
*C. elegans: yphEx350[flp-24p::FLP::unc-54 3′UTR +unc-122p::GFP::unc-54 3′UTR]; let-23(zh131[FRT::let-23::FRT::GFP::LoxP::FLAG::let-23]) II.*	This paper	TWN5107
*C. elegans: yphEx351[flp-11p::FLP::unc-54 3′UTR +unc-122p::GFP::unc-54 3′UTR]; let-23(zh131[FRT::let-23::FRT::GFP::LoxP::FLAG::let-23]) II.*	This paper	TWN5108

**Recombinant DNA**		

Plasmid: let-23p(2kb):GCaMP6s:unc-54 3′ UTR	This paper	pPH955

**Software and algorithms**		

Graphpad Prism 9	GraphPad	https://www.graphpad.com
RStudio	RStudio PBC	https://posit.co/products/open-source/rstudio/
Tracker	Open Source Physics	https://www.physlets.org/tracker/
FIJI	Schindelin et al.^46^	https://fiji.sc

### Experimental model and study participant details

#### Strains and culture conditions

The wild-type strain used in this study was *C. elegans* strain CGC1. Worms were cultured at 23°C on nematode growth medium (NGM) plates seeded with *E. coli* OP50 as a food source. All experiments utilized adult hermaphrodite worms. Transgenic strains used in this study are listed in the key resources table. The fungal strain *Arthrobotrys oligospora* TWF154 was cultured on potato dextrose agar (PDA, i.e., rich-nutrient) plates at 25°C for maintenance. All experiments were conducted using a low-nutrient medium (LNM) containing 2% agar, 1.66 mM MgSO_4_, 5.4 μM ZnSO_4_, 2.6 μM MnSO_4_, 18.5 μM FeCl_3_, 13.4 mM KCl, 0.34 μM biotin, and 0.75 μM thiamine, at 23°C.

### Method details

#### Arthrobotrys oligospora trap induction

To induce trap formation, *A. oligospora* TWF154 was initially cultured on PDA plates. A 3-mm PDA square containing fungal culture was chunked and transferred to LNM and grown at 25°C for 5 days to pre-starve the fungus. A subsequent 3-mm square from the pre-starved LNM culture was transferred to fresh LNM and incubated for 4 days at 25°C. By day 4, fungal hyphae reached the edge of the 5-cm plates. Approximately 300–500 young adult/adult *C. elegans* were added to the plates and incubated overnight at 25°C to induce traps. All experiments were performed using these trap-induced cultures on day 5.

#### Quantifying pharyngeal pumping

Adult hermaphrodite worms were washed in M9 buffer and allowed to settle for 1–2 min by gravity to minimize handling stress. Approximately 100 worms were transferred to *A. oligospora* trap-induced plates. Trapping typically occurred within a few minutes. Pharyngeal pumping was observed under a stereo-dissecting microscope for 15 s every 5 min. Pumping rates were calculated as pumping events per second (Hz).

#### Tracking head movements and neuron movements

Adult hermaphrodite worms were prepared as described above and placed on LNM plates. Fluorescently labeled pharyngeal muscles were used to enhance image contrast for automated tracking. Time-lapse videos were captured with a stereo-dissecting microscope at 6 frames per minute over 1 h with continuous GFP excitation. Movements were analyzed using Tracker (Open Source Physics) with default autotracker settings and manually curated when necessary.

#### Head-drop avoidance assay

To assess glycerol avoidance behavior, a 0.5M glycerol drop was applied to worms using a capillary tube. For active worms, the glycerol drop was placed in front of forward-moving individuals, and backward movement was scored as avoidance. For quiescent worms, the drop was placed just ahead of their position. The fraction of worms exhibiting avoidance was calculated as the number of backward responses divided by the total number tested. Each data point represents 10 worms.

#### Neuronal calcium imaging

Calcium levels in ALA (*let-23p::GCaMP*) and RIS (*flp-11p::GCaMP*) neurons were monitored using GCaMP. Worms trapped by *A. oligospora* were chunked into a 3-cm LNM square, inverted, and mounted on a coverslip. Imaging was performed using a Zeiss AxioObserver.D1 or Z1 microscope with either a Photometrics Evolve 512 EMCCD or Prime BSI CMOS camera, capturing frames at 1 Hz for 60 min. Fluorescence intensities were analyzed using Fiji, normalized as (F−F_0_)/F_0_, where F_0_ is the baseline fluorescence averaged over the first minute of recording.

#### WormGlu physical restriction

Worms were immobilized using WormGlu (GluStitch Inc., Delta, Canada) following a brief 3-min cooling on ice. Once immobile, WormGlu was applied for 2 min using a hand-pulled capillary. Immobilized worms were recovered at 23°C for 5 min before scoring pharyngeal pumping rates over 30 min.

### Quantification and statistical analysis

Statistical analyses and visualization were performed in GraphPad Prism 9 or R version 4.3.0. Test used were described in the figure legends with sample sizes indicated.

## Supplemental information

Document S1. Figures S1 and S2

Video S1. *C. elegans* response to *A. oligospora* predation from struggling to quiescence, related to Figure 1 This time-lapse video showed the *C. elegans* struggling in the first 20 min before entering a quiescent state. Time codes at the upper left indicate hh:mm:ss.
