# Supplementary material for: Predation by nematode-trapping fungus triggers mechanosensory-dependent quiescence in Caenorhabditis elegans
Source: iScience. 2025 May 30;28(7):112792. doi: 10.1016/j.isci.2025.112792 (PMC12205622; doi:10.1016/j.isci.2025.112792)
Supplement: Document S1. Figures S1 and S2 [file mmc1.pdf]

iScience, Volume *28*

Supplemental information

Predation by nematode-trapping fungus triggers mechanosensory-dependent quiescence in *Caenorhabditis elegans*

Tzu-Hsiang Lin, Han-Wen Chang, Rebecca J. Tay, and Yen-Ping Hsueh

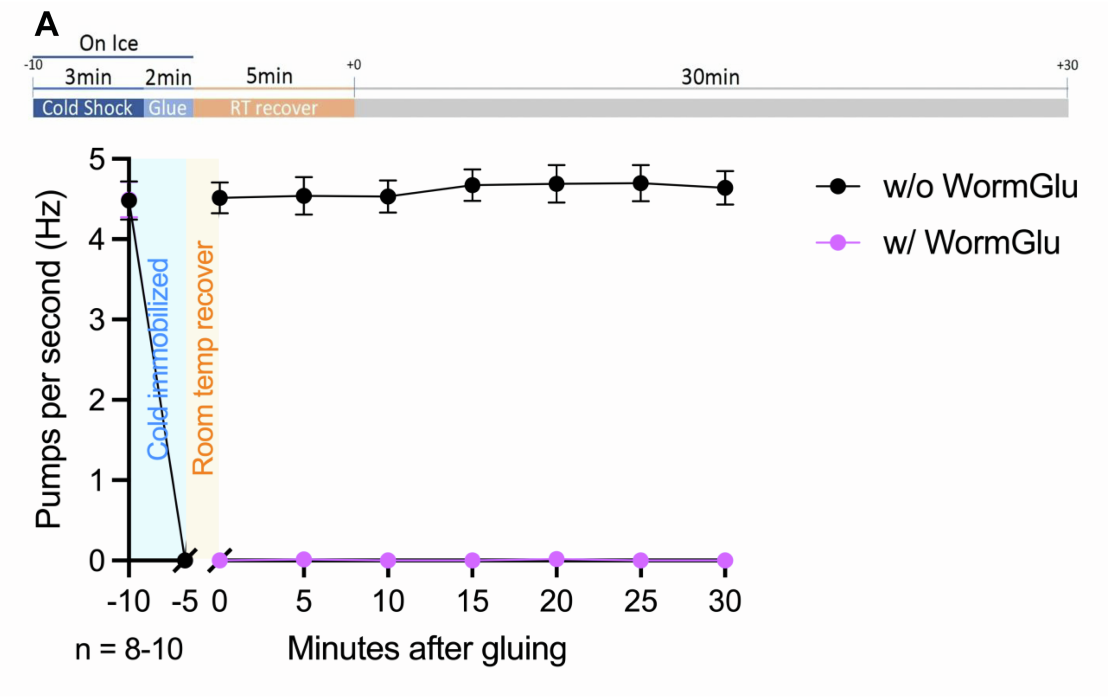

Figure S1. WormGlu adhesion-induced pumping quiescence, Related to Figure 3

(A) Schematic illustration of the experimental procedure used to immobilize worms using WormGlu (top) and traces showing pharyngeal pumping rates in worms with and without WormGlu adhesion (bottom).

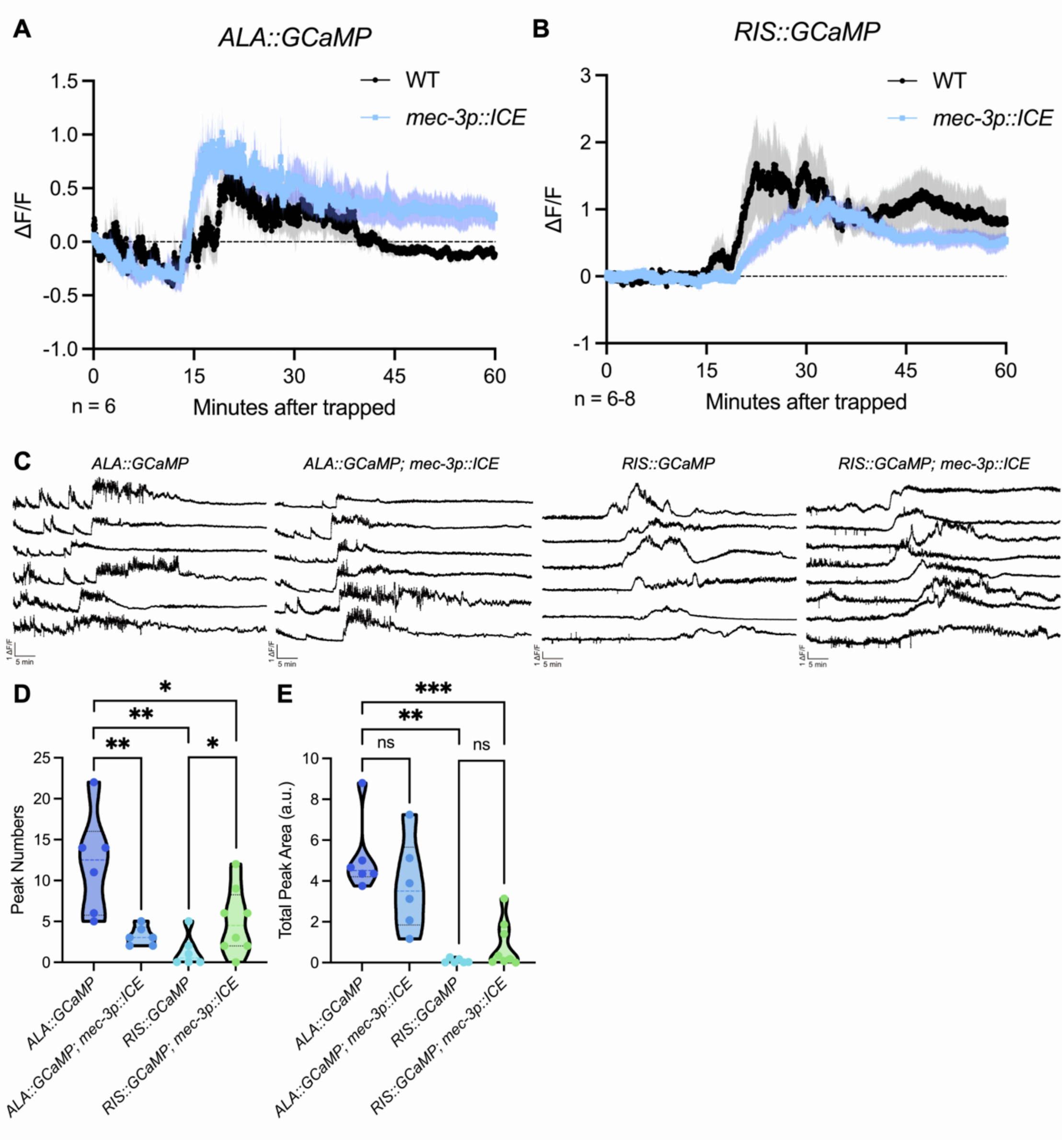

Figure S2. Mechanosensory pathways involved in sleep-promoting neuron activity patterns, Related to Figure 3

(A) Average calcium activity traces of ALA neurons in wild-type and genetically ablated mechanosensory mutants after *A. oligospora* trapping. Mean ± SEM.

(B) Average calcium activity traces of RIS neurons in wild-type and genetically ablated mechanosensory mutants after *A. oligospora* trapping. Mean ± SEM.

(C) Individual calcium activity traces from panels A and B show transient peak activation of ALA neurons during the early phase.

(D) Number of calcium activity peaks in individual experiments during the first 20 minutes, defined as events exceeding 10% of the minimum-to-maximum amplitude and lasting more than 10 seconds. Mann–Whitney test; *$p < 0.05$, **$p < 0.01$.

(E) Area under the curve of calcium activity peaks in individual experiments during the first 20 minutes, using the same criteria as in panel D. Mann–Whitney test; $^{**}p < 0.01$, $^{***}p < 0.001$.
